# A Comparative Biochemical Study of Oleate Hydratases

**DOI:** 10.1002/cbic.70487

**Published:** 2026-07-30

**Authors:** Maxim van Delft, Alejandro Gran‐Scheuch, Ulf Hanefeld, Peter‐Leon Hagedoorn

**Affiliations:** ^1^ Department of Biotechnology Delft University of Technology Delft The Netherlands

**Keywords:** biocatalysis, enzymology, fatty acids, oleate hydratase, reducing conditions

## Abstract

In the search for more efficient chemical processes to valorize renewable feedstocks, oleate hydratases (Ohys) represent promising biocatalysts. These enzymes can convert *cis*‐Δ^9^ fatty acids like oleic acid into their hydroxy fatty acid counterparts using only water as co‐reagent and with unparallelled selectivity. A multitude of Ohys has been described, however, direct comparison is hard due to the use of different assays. For this work, we characterized and compared four Ohys across different homologous families (HFams): those from *Elizabethkingia meningoseptica* (*Em*Ohy, HFam11), *Stenotrophomonas nitritireducens* (*Sn*Ohy, HFam11), *Rhodococcus erythropolis* (*Re*Ohy, HFam3), and *Rhodococcus pyridinivorans* (*Rp*Ohy, HFam2). All enzymes, especially *Sn*Ohy, expressed well in *Escherichia coli* (28–99 mg/L_culture_). FAD occupancy after purification was 51% or lower for all Ohys. *Re*Ohy had lost the flavin altogether, making supplementation a necessity for its activity. Reduction of the cofactor to FADH_2_ clearly showed a favorable effect on the activity of all Ohys, with product yields increasing from modest to 12‐fold. Although *Sn*Ohy showed the highest initial reaction rates, *Em*Ohy reached the highest 10‐hydroxystearic acid (10‐HSA) yields at higher oleic acid loading (10 mM) and prolonged reaction times. Thermostability was analyzed by thermal shift assays, with *Em*Ohy appearing as most thermostable (*T*_*m*_^app^ = 54 °C).

## Graphical abstract

Four oleate hydratases (Ohys) from diverse homologous families (HFams) were characterized side‐by‐side in terms of expression levels, oligomeric state, flavin adenine dinucleotide (FAD) affinity, thermostability, and activity. A general favorable effect of the reduction of FAD to FADH_2_ for Ohy activity was demonstrated, with (*R*)‐10‐hydroxystearic acid (10‐HSA) yield increasing up to 12‐fold.

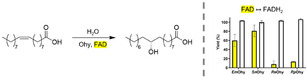

## 1 Introduction

**SCHEME 1:**
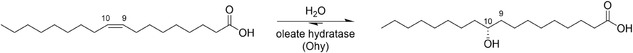
The conversion of oleic acid to (*R*)‐10‐hydroxystearic acid by oleate hydratase.

A greener chemical industry that is less reliant on fossil resources requires improved ways of valorizing renewable feedstocks like vegetable oils [1]. In this context, oleate hydratases (Ohys, EC 4.2.1.53) are promising biocatalysts. Whereas oleochemistry classically has a focus on transformation of the carboxyl group, these enzymes hydrate the double bond of several *cis*‐Δ^9^ fatty acids to form their hydroxy fatty acid counterpart, primarily oleic acid to (*R*)‐10‐hydroxystearic acid (10‐HSA, Scheme 1) [2, 6]. The complete regio‐ and near‐perfect stereoselectivity of the reaction remains unattainable with other chemical strategies [7]. Hydroxy fatty acids have useful properties for, among others, surfactant, emollient, and lubricant applications, and DSM‐Firmenich already employs oleate hydratases for the commercial production of 10‐HSA as a cosmetic ingredient [8].

Due to their interesting reactivity and practical potential, the discovery, characterization, and engineering of oleate hydratases are increasingly the subject of research [9, 13]. To date, the crystal structure of five different Ohys has been reported [14, 18]. Interestingly, all Ohys require a flavin adenine dinucleotide (FAD) cofactor for activity, although its redox state is expected to remain unchanged during catalysis, as hydration is a redox‐neutral reaction. The exact role of FAD remains elusive. No indications were found for involvement in protein stabilization or direct participation in the reaction mechanism but an intricate role in repositioning amino acids into the catalytic conformation has been proposed [15, 18]. The FAD affinity of oleate hydratases is modest, and the cofactor is lost to a great extent during purification [15, 16, 19]. Interestingly, a clear activity‐enhancing effect was observed by chemical reduction of the flavin for multiple Ohys, and it is hypothesized to be reduced under physiological conditions in vivo [15, 20, 21].

Based on sequence identity, Ohys are classified into 11 different homologous families (HFams) as established in the Hydratase Engineering Database (HyED), with HFam2, 3, and 11 containing the currently most prominent Ohys [22]. For instance, the oleate hydratase from *Elizabethkingia meningoseptica* (*Em*Ohy, HFam11) was the first Ohy of which the coding gene was discovered and has been extensively characterized [15, 23]. A reaction mechanism was proposed with a crucial role for a glutamate (E122) and a tyrosine (Y241) residue: for activation of a water molecule and protonation of the double bond, respectively. With respect to oligomeric state, HFam11 Ohys have been described as homodimers in solution; a large part of the dimeric interface is made up of N‐ and C‐terminal extension loops. HFam2 is the largest family and includes Ohys from *Lactobacillus acidophilus* (*La*Ohy) and *Rhodococcus pyridinivorans* (*Rp*Ohy) [14, 24]. Previous analyses indicated the occurrence of both dimeric and monomeric forms of these enzymes. Lastly, HFam3 Ohys are clearly distinct from the enzymes in HFam2 and 11. The oleate hydratase from *Rhodococcus erythropolis* (*Re*Ohy) can be considered a model enzyme for this family [16]. More recently, an Ohy from *Pediococcus parvulus* (*Pp*Ohy) was added [19]. Both occur only as a monomer in solution, presumably because of the lack of N‐ and C‐terminal extensions. Also, in terms of mechanism, HFam3 distinguishes itself from HFam2 and 11. Instead of the catalytic glutamate, HFam3 Ohys contain a methionine at the corresponding position (e.g., M77 in *Re*Ohy), strongly suggesting a different catalytic mechanism. Additionally, the FAD affinity seems distinct; while Ohys from HFam2 and 11 show the presence of FAD after purification, HFam3 Ohys are apo‐enzymes upon isolation.

Still, more characterization data remains desirable, and a direct comparison of different Ohys based on literature data is challenging due to different methods of analysis and conditions used. In particular, activity is difficult to compare, even when kinetic data are obtained with purified enzymes. Partially, this is due to the hydrophobic nature of fatty acids, which strongly affects substrate availability. Hence, activity is highly dependent on, for instance, substrate and cosolvent concentration, pH, and mixing speed.

Thus, to obtain a more comprehensive understanding of Ohys and directly compare their properties, we selected four different oleate hydratases for characterization under identical conditions. The selection comprises *Em*Ohy and *Re*Ohy, the Ohy from *Stenotrophomonas nitritireducens* (*Sn*Ohy, HFam11), and *Rp*Ohy. *Sn*Ohy was added due to its exceptionally high reported turnover number (Table 1) [25] and *Rp*Ohy to include an Ohy from HFam2. Although not fully doing justice to the complete diversity of the homologous families, this selection of enzymes represents a diverse Ohy subset. The enzymes were heterologously expressed and characterized, in particular, aiming to better understand their relative activities and the role of FAD.

**TABLE 1:** Oleate hydratases selected for this study, with corresponding homologous family, reported activity, effect of FAD reduction on activity, and PDB entry. The amino acid sequence identity matrix of these enzymes is presented in Table S1.

Organism	Ohy	HFam	Oligomeric state	*k*_*cat*_, s^−1^	Effect of FAD reduction	PDB entry	References
*Elizabethkingia meningoseptica*	*Em*Ohy	11	Dimer	1.2	7‐fold increase	4UIR	[15, 23]
*Stenotrophomonas nitritireducens*	*Sn*Ohy^a^	11	Dimer	78	n.d.^b^	5Z70	[17, 25, 26]
*Rhodococcus erythropolis*	*Re*Ohy^c^	3	Monomer	0.57	n.d.^b^	5ODO	[16]
*Rhodococcus pyridinivorans*	*Rp*Ohy	2	Monomer and dimer	20	n.d.^b^	n.a.	[24]

a The crystal structure was reported for the oleate hydratase from *Stenotrophomonas* sp. *KCTC 12332* (accession number AMJ57405.1), but its sequence is identical to that of *Sn*Ohy (AND01240.1).

b n.d.: not determined.

c While most work on *Re*Ohy concerns *Re*Ohy_CCM2595_, this study uses *Re*Ohy (PR4) as in our previous report. Sequences differ by only three amino acids [16, 27, 29].

## 2 Results and Discussion

### 2.1 Expression and Oligomeric State Analysis

The N‐terminally His‐tagged oleate hydratases were expressed in *Escherichia coli* (*E. coli*) and purified by immobilized metal affinity chromatography (IMAC) in good yield, generally tens of mg per liter of cell culture (Figure S1, Table S2). Especially for *Sn*Ohy, high expression yields were obtained (99 mg/L_culture_). Surprisingly, upon codon optimization of the gene and use of a high‐level protein expression system (pET in BL21(DE3) instead of pBAD in TOP10), *Em*Ohy yield dropped from 28 to 9 mg/L_culture_.

**FIGURE 1:**
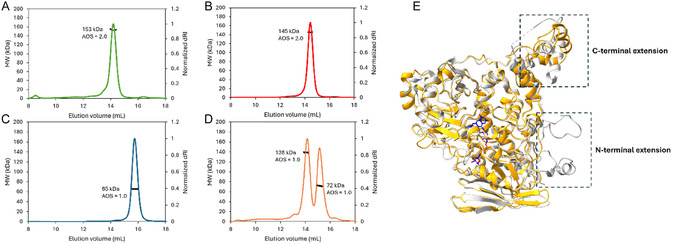
SEC‐MALS analysis of the oleate hydratases and structural explanation of the monomer‐dimer equilibrium of *Rp*Ohy. (A–D) show the chromatograms, in the order *Em*Ohy, *Sn*Ohy, *Re*Ohy and *Rp*Ohy. The calculated molecular weights are given with their corresponding peaks, along with the corresponding apparent oligomeric state (AOS). (E) shows an overlay of *Rp*Ohy (orange, AlphaFold2 model) and chain A of *Em*Ohy (gray, PDB: 4UIR). The boxes indicate the N‐ and C‐terminal extensions of *Em*Ohy, together making up a major part of the dimerization interface. Figure made with ChimeraX.

The oligomeric state of the Ohys was determined by size‐exclusion chromatography coupled to multi‐angle light scattering (SEC‐MALS). Consistent with earlier reports, the HFam11 enzymes *Em*Ohy and *Sn*Ohy were observed as dimers, and *Re*Ohy as a monomer (Figure 1A–C) [15, 16, 25]. *Rp*Ohy occurs both in the dimeric and monomeric form, in an approximate 1:1 ratio (Figure 1D), as also indicated before [24]. This co‐existence of both oligomeric states may be rationalized based on sequence and structural analysis. In HFam11 Ohys, a large part of the dimeric interface is made up of both a C‐terminal and an N‐terminal extension. While *Rp*Ohy does have a C‐terminal extension, it lacks the long N‐terminal extension (Figures 1E, S2), thus occurrence as both dimer and monomer in equilibrium is reasonable [30].

### 2.2 FAD Occupancy

**FIGURE 2:**
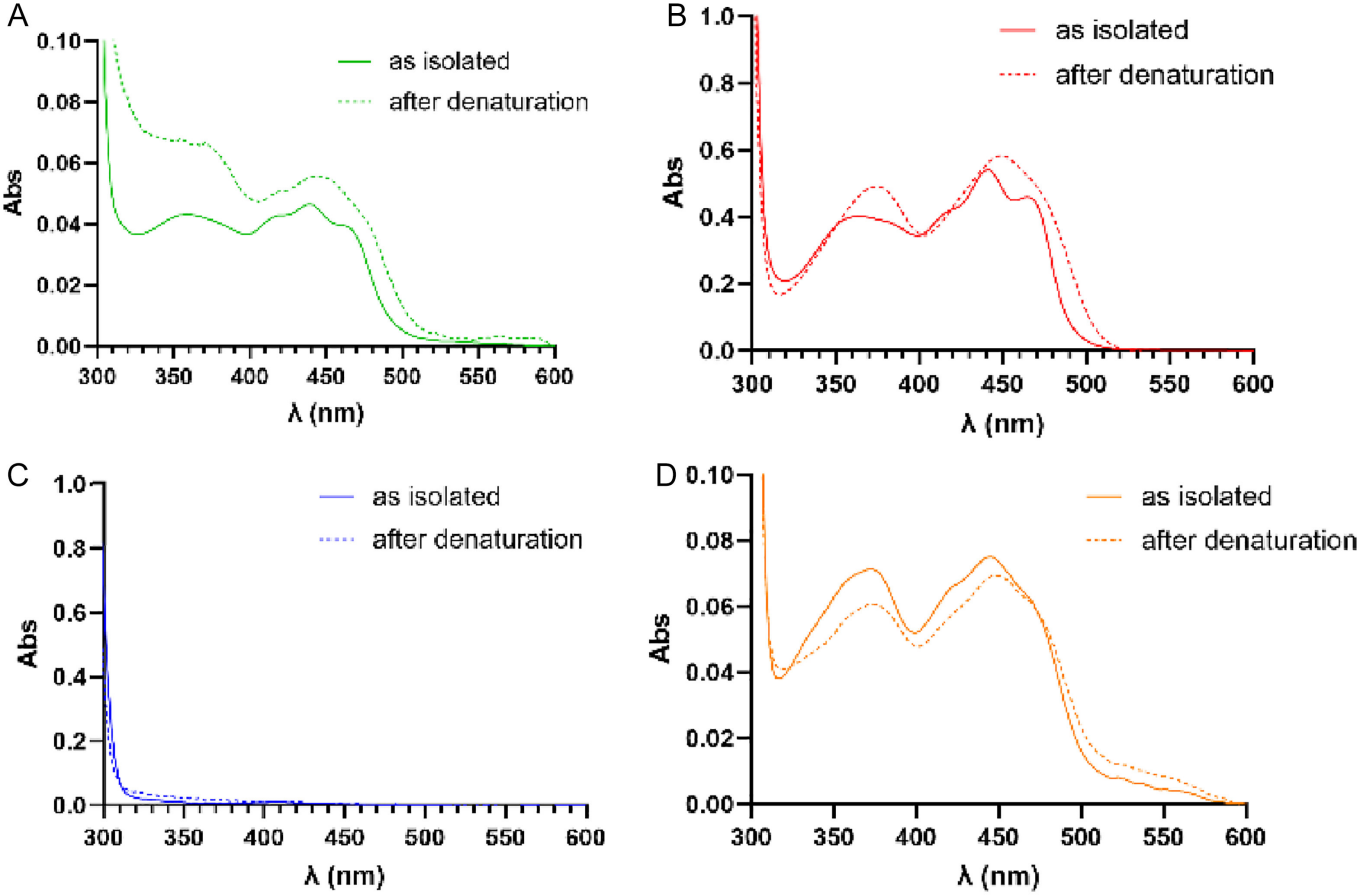
UV–Vis absorption spectra of the oleate hydratases before and after denaturation: (A) *Em*Ohy (17 µM), (B) *Sn*Ohy (104 µM), (C) *Re*Ohy (72 µM) and (D) *Rp*Ohy (39 µM).

Purified *Em*Ohy, *Sn*Ohy, and *Rp*Ohy stocks appeared pale yellow while *Re*Ohy was always colorless, even when FAD was supplemented during cell lysis prior to purification. Indeed, only the former three Ohys showed a UV–Vis spectrum characteristic of flavoenzymes (Figure 2). The FAD occupancy after purification was determined by denaturing the enzyme and determining the free FAD concentration based on the absorbance at 450 nm. *Sn*Ohy showed the highest FAD occupancy at 51%, followed by *Em*Ohy (30%) and *Rp*Ohy (16%). The substoichiometric FAD occupancy does not seem to be a consequence of insufficient intracellular FAD concentration during expression, as the best‐expressed Ohy (*Sn*Ohy) also shows the highest FAD occupancy. As expected, no FAD‐typical absorbance peak was observed for *Re*Ohy, confirming the loss of its cofactor during purification and thus a lower FAD affinity compared to the other Ohys (Table 2).

**TABLE 2:** FAD occupancies of the oleate hydratases after purification.

Ohy	FAD occupancy, %
*Em*Ohy	30
*Sn*Ohy	51
*Re*Ohy	n.d.^a^
*Rp*Ohy	16

a n.d.: not determined due to absence of *A*_450_ peak.

### 2.3 Relative Activities and Attempted Reconstitution of FAD

**FIGURE 3:**
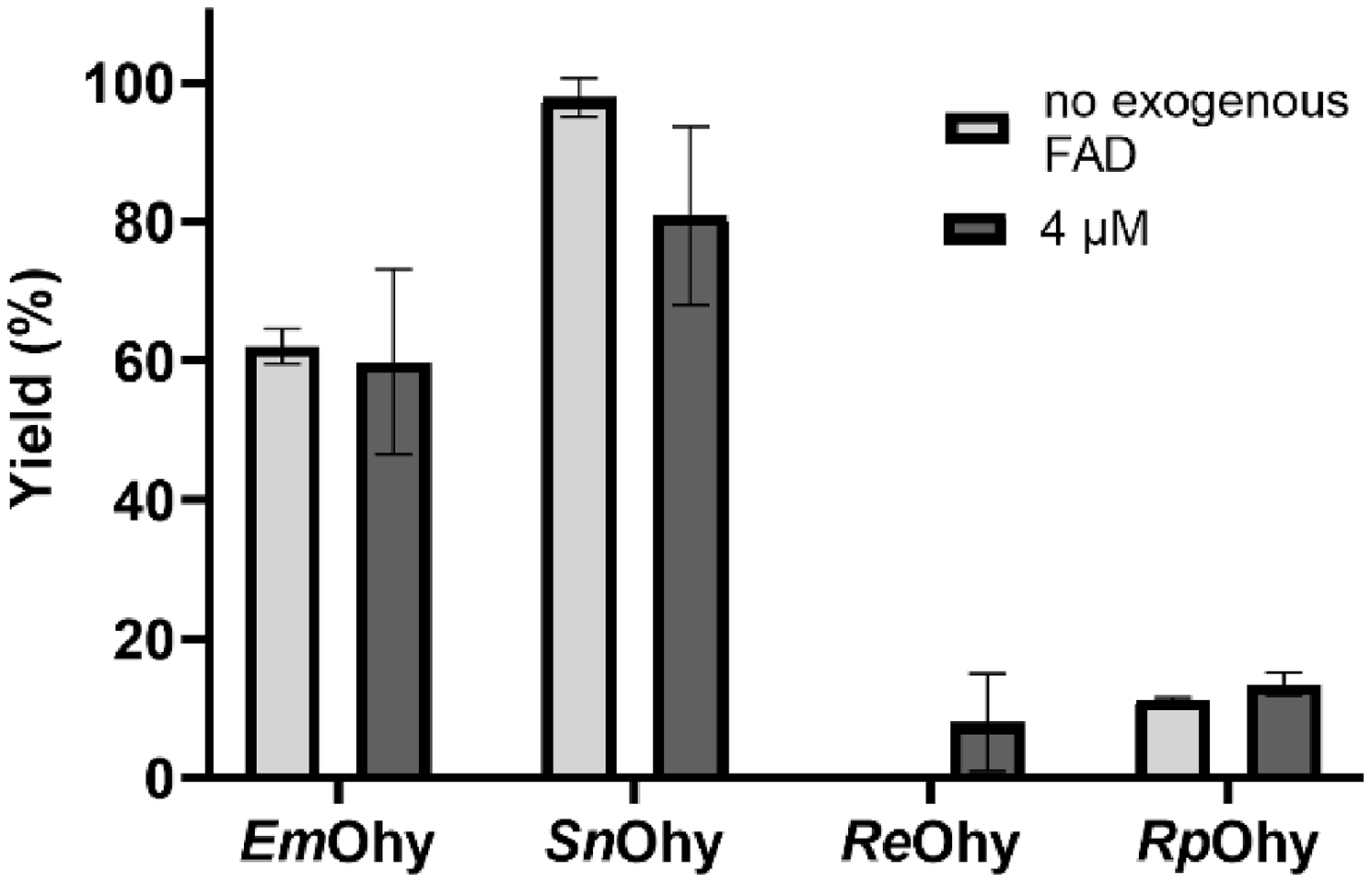
Effect of FAD supplementation on product yields of the Ohys in biotransformations. Reaction conditions: 1.0 mM OA, 1.0 µM Ohy, 0 or 4.0 µM FAD, 5.0% v/v DMSO, in 100 mM KP_i_ 7.0, total volume 500 µL; 25 °C, 1000 rpm orbital shaking, reaction time 10 min. Each bar represents the mean of triplicates, and the error bars represent their standard deviation.

The activities of the Ohys were assessed with short (10‐minute) reactions with 1 mM oleic acid (OA) concentration. *Sn*Ohy reached the highest product concentrations, followed by *Em*Ohy and then the *Rhodococcus* enzymes (Figure 3). Because the Ohys occurred partially in their apo‐form, FAD was supplemented during the biotransformations. However, for *Em*Ohy, *Sn*Ohy, and *Rp*Ohy, 10‐HSA yields remained unaltered upon moderate addition (4:1 molar ratio to Ohy) of FAD compared to control reactions without exogenous FAD. Only for *Re*Ohy, which was isolated as an apo‐enzyme after purification, FAD supplementation was shown to be essential for activity. Even higher concentrations and pre‐incubation with FAD did not increase activity (Figure S3). The lack of effect of FAD supplementation on Ohy activity was surprising, since the concentrations were well above the reported dissociation constant of *Em*Ohy (*K*_*D*_ = 1.8 µM) [15]. It is considered unlikely that flavin‐deficient Ohys are active, based on earlier studies with *Em*Ohy and *Re*Ohy [15, 29], and on the data presented here for *Re*Ohy. However, our data indicate that reconstitution of the cofactor in already folded oleate hydratase is not trivial.

**FIGURE 4:**
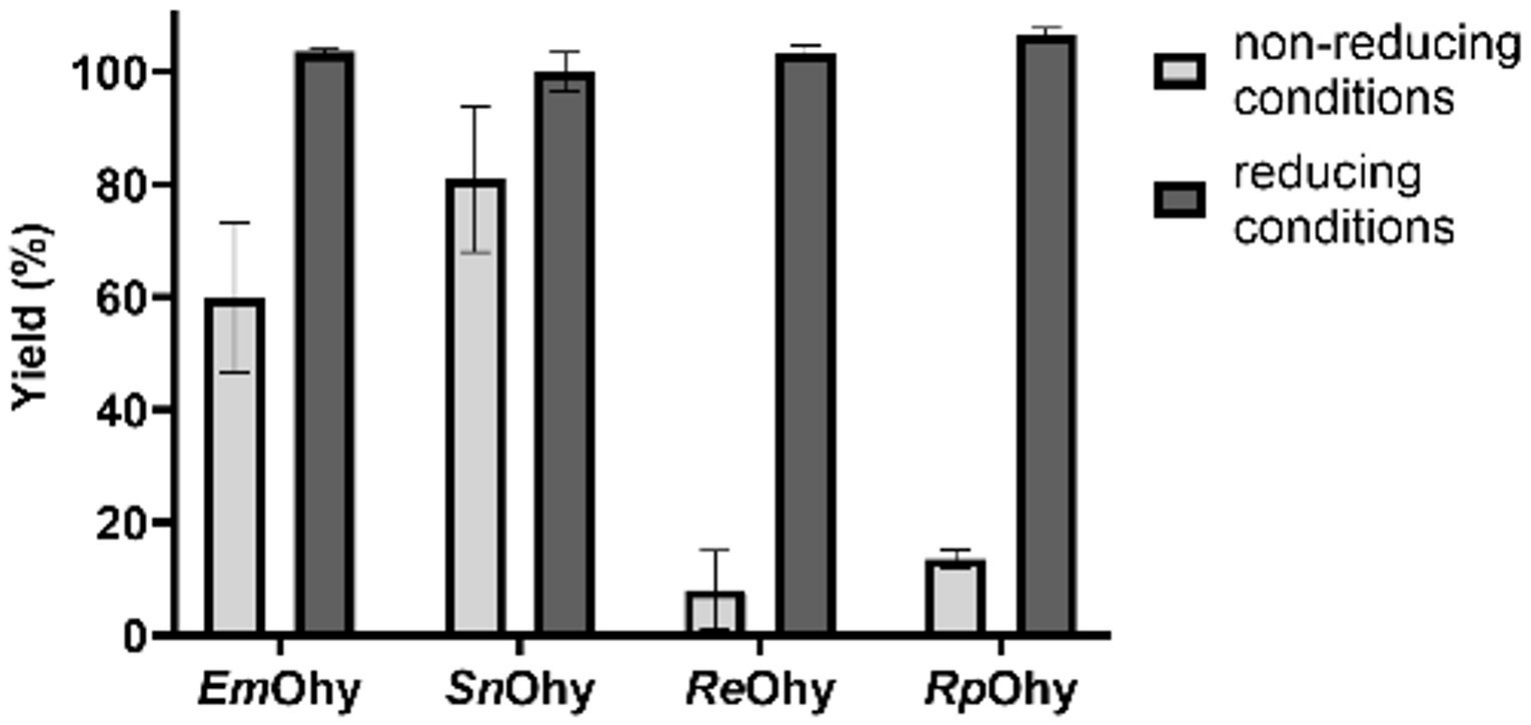
Effect of reducing conditions on the product yields of the oleate hydratases in biotransformations at 1.0 mM oleic acid loading. Reaction conditions: 1.0 mM OA, 1.0 µM Ohy, 4.0 µM FAD, 5.0% v/v DMSO in 100 mM KP_i_, pH 7; 25 °C, 1000 rpm, 10 min reaction time. For reducing conditions, 2.0 mM dithionite was added, and the reaction was performed anaerobically. Each bar represents the mean of triplicates, and error bars are their standard deviation [23, 29].

To better understand the role of FAD, and because earlier studies indicated a large effect of reduction of FAD to FADH_2_ on Ohy activity [15, 20, 21], similar reactions were conducted under reducing conditions. Interestingly, under these conditions, all enzymes, including the less active *Rhodococcus* Ohys, reached full conversion (Figure 4). For *Re*Ohy, this corresponds to at least a 12‐fold increase in activity. The clear activity‐enhancing effect of FAD supplementation under reductive conditions, in contrast to the above‐described results, may indicate an important role for the conformation of the isoalloxazine ring. Upon reduction, the FAD undergoes a conformational shift from a planar, aromatic structure to a bent “butterfly” shape [31]. However, this remains speculative, and detailed biophysical analyses would be required to further investigate this hypothesis.

**FIGURE 5:**
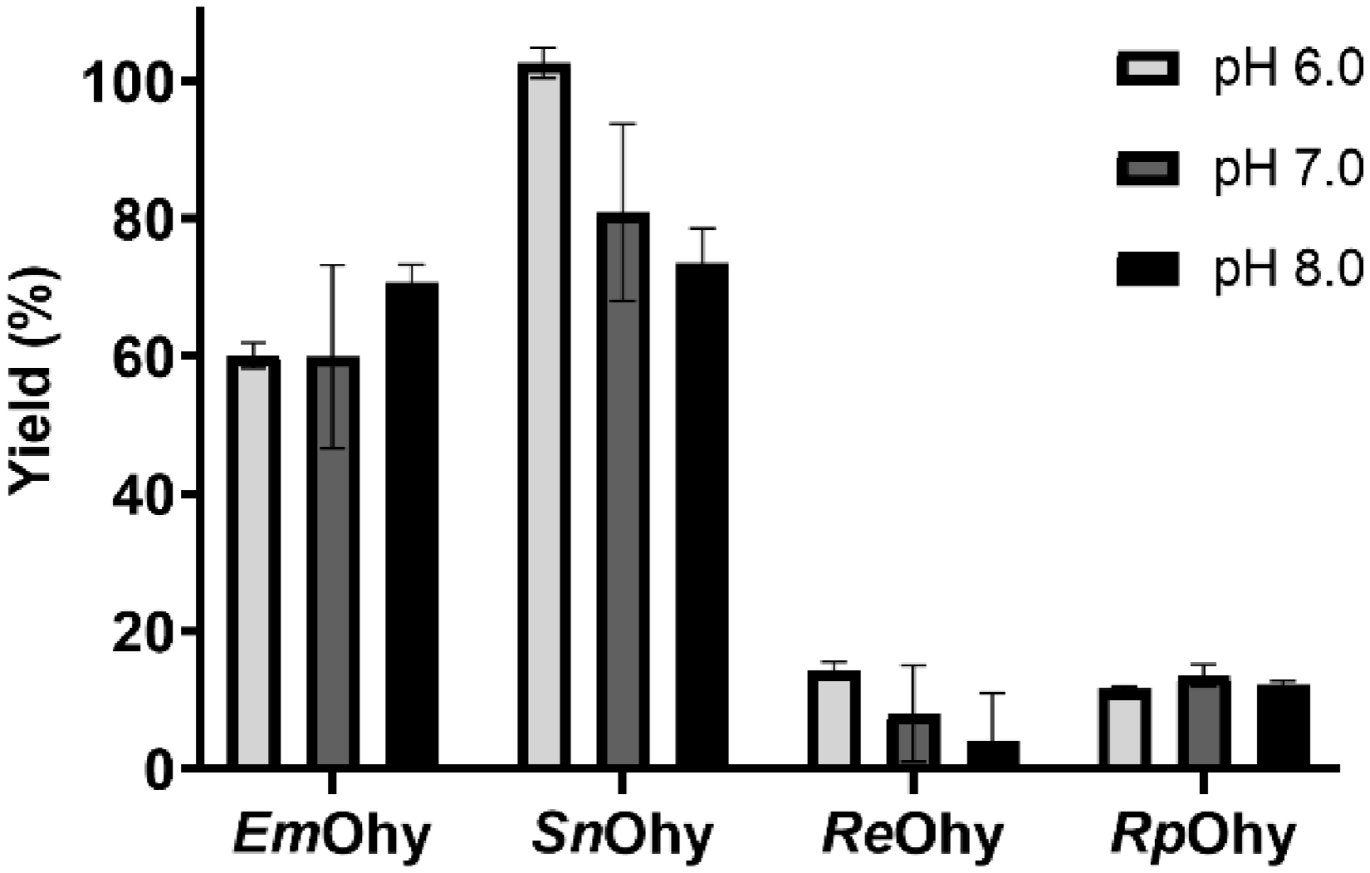
Effect of pH on the product yields of the Ohys in biotransformations. Reaction conditions: 1.0 mM OA, 1.0 µM Ohy, 4.0 µM FAD, 5.0% v/v DMSO, in 100 mM KP_i_ pH 6.0, 7.0 or 8.0, total volume 500 µL; 25 °C, 1000 rpm orbital shaking, reaction time 10 min. Each bar represents the mean of triplicates, and error bars represent their standard deviation.

To investigate differences in pH preference among the Ohys, reactions were conducted at varying pH values besides pH 7.0. Also at pH 6.0 and 8.0, the highest product formation was achieved in the order of *Sn*Ohy, *Em*Ohy, *Rp*Ohy, and *Re*Ohy (Figure 5). *Em*Ohy and *Rp*Ohy show relatively consistent activity across the different pHs; for *Sn*Ohy and *Re*Ohy, pH 6.0 is preferred. The observed rate of *Em*Ohy at pH 6.0 (*k*_obs_ = 1.0 s^−1^) is in the same range as its reported *k*_cat_ of 1.2 s^−1^ under similar conditions [15]. Observed activities of *Sn*Ohy and *Rp*Ohy are not as high as perhaps would be expected based on their reported *k*_cat_ values. For instance, the *k*_obs_ values of *Sn*Ohy (1.5 s^−1^) and *Rp*Ohy (0.24 s^−1^) at pH 7.0 are much lower than their reported turnover numbers of 78 and 20 s^−1^, respectively [24, 25]. However, the *k*_obs_ and *k*_cat_ values should be compared with caution, as differences in assay conditions are to be considered. The observation that HFam11 Ohys exhibit a relatively high activity is consistent with earlier reports [12, 22].

### 2.4 Enzyme Stability

As an indicator of enzyme thermostability, the apparent melting temperatures of the Ohys were determined by thermal shift assays using both the ThermoFluor and ThermoFAD method [32, 33]. These assays follow protein unfolding by gradually increasing the temperature, with the melting temperature representing the inflection point of the transition from folded to fully unfolded protein. However, the ThermoFluor and ThermoFAD assays apply different indicators. The ThermoFluor assay utilizes a dye that binds to the hydrophobic parts of proteins, which become exposed during denaturation. Upon binding to these hydrophobic regions, the dye emits a fluorescent signal. ThermoFAD is a similar approach that works only for flavoproteins by monitoring the increase in fluorescence resulting from FAD release during denaturation. Although the methodologies slightly differ, the measured values were averaged, as they determine the same property (apparent melting temperature) under very similar conditions. These ranged from 36.0 °C for *Re*Ohy to 53.8 °C for *Em*Ohy (Table 3, Figure S4). For *Em*Ohy, the value is well in line with an earlier report; for *Re*Ohy, it is substantially lower than the earlier reported 45 °C [19]. Differences in buffer type and concentrations of protein and dye are likely to contribute to this. The inability to obtain a melting curve of *Re*Ohy by ThermoFAD is explained by the absence of the cofactor. As suggested before, the higher stability of *Em*Ohy versus monomeric *Re*Ohy could possibly be attributed to stabilization conferred by its protein–protein interface. However, a general stabilizing effect of dimerization is not evident from this study: dimeric *Sn*Ohy has a lower apparent melting temperature (41.5 °C) than partially monomeric *Rp*Ohy (47.9 °C).

**TABLE 3:** Apparent melting temperatures of the oleate hydratases.

Ohy	*T*_*m*_^app^, °C		
	ThermoFluor	ThermoFAD	Average
*Em*Ohy	53.7 ± 0.1	53.9 ± 0.3	53.8 ± 0.3
*Sn*Ohy	41.2 ± 0.6	41.7 ± 0.8	41.5 ± 1.0
*Re*Ohy	36.0 ± 0.3	n.d.^a^	36.0 ± 0.3
*Rp*Ohy	43.4 ± 0.3	52.4 ± 0.6	47.9 ± 0.6

a n.d.: not detected.

### 2.5 Activity at Higher Substrate Loading

**FIGURE 6:**
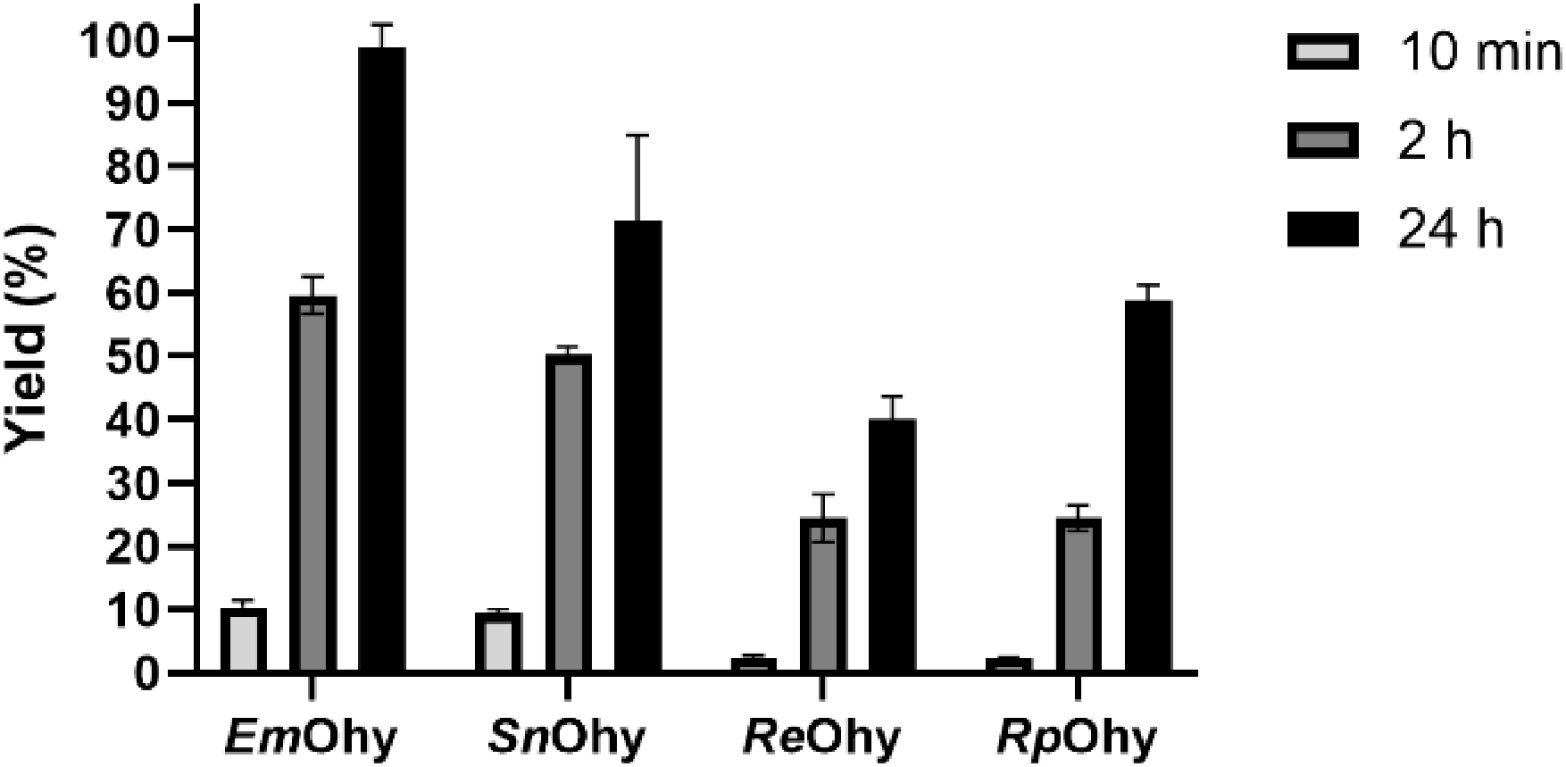
Time‐dependent product yields of the Ohys in biotransformations at higher oleic acid loading. Reaction conditions: 10 mM OA, 1.0 µM Ohy, 4.0 µM FAD, 5.0% v/v DMSO, in 50 mM PIPES, pH 7.0, total volume 500 µL; 25 °C, 1000 rpm orbital shaking, reaction time 10 min, 2, or 24 h. Each bar represents the mean of triplicates, and error bars represent their standard deviation.

To compare the performance of the different Ohys under more process‐like conditions, reactions were conducted at increased OA loading (10 mM). Reactions were conducted for 10 min, 2, and 24 h (Figure 6). Under these conditions, substrate saturation is achieved for all Ohys: the concentration is well above the *K*_*M*_ values of *Em*Ohy (110 µM), *Sn*Ohy (22 µM), *Re*Ohy (490 µM), and *Rp*Ohy (720 µM) [3]. *Em*Ohy showed the highest product formation, also compared to *Sn*Ohy, being the only enzyme to reach full conversion in 24 h. This implies that *Em*Ohy is active for longer due to higher intrinsic stability, which is consistent with its relatively high apparent melting temperature. Plausibly, enzyme deactivation occurs within 24 h, at least for *Sn*Ohy, *Re*Ohy, and *Rp*Ohy. Although substrate inhibition has not been observed for Ohys [3], the high concentration of OA may cause enzyme deactivation, given its highly apolar character.

**FIGURE 7:**
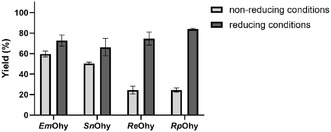
Effect of reducing conditions on the product yields of the oleate hydratases in biotransformations at 10 mM oleic acid loading. Reaction conditions: 10 mM OA, 1.0 µM Ohy, 4.0 µM FAD, 5.0% DMSO in 100 mM KP_i_, pH 7; 25 °C, 1000 rpm, 2 h reaction time. For reducing conditions, 2.0 mM dithionite was added, and the reaction was performed anaerobically. Each bar represents the mean of triplicates, and error bars represent their standard deviation.

Like for the 1 mM substrate reactions, reducing conditions improved the activity of all Ohys in reactions with 10 mM OA (Figure 7). However, the effect is much smaller, and product yield plateaued around 80%. Again, this may indicate enzyme inactivation over time, caused or amplified by the presence of the reducing agent dithionite. Alternatively, under these conditions, it may not be the concentration of active Ohy that is the rate‐limiting factor, but instead the availability of free OA molecules to the enzyme. Above the solubility limit, OA occurs as micelles, vesicles, or other structures that may render it less accessible for the reaction [34].

## 3 Conclusion

This work presented a comparative biochemical study of four oleate hydratases across different HFams: those from *Elizabethkingia meningoseptica* (*Em*Ohy, HFam11), *Stenotrophomonas nitritireducens* (*Sn*Ohy, HFam11), *Rhodococcus erythropolis* (*Re*Ohy, HFam3), and *Rhodococcus pyridinivorans* (*Rp*Ohy, HFam2). All enzymes were expressed in *E. coli* and obtained in good yield, especially *Sn*Ohy (99 mg/L_culture_). Oligomeric states were in line with the literature, including the occurrence of both monomeric and dimeric forms of *Rp*Ohy. This was explained based on the presence of the C‐terminal extension but the absence of the N‐terminal extension of *Rp*Ohy, with both making up a major part of the interface of dimeric Ohys. All Ohys had substoichiometric FAD content after purification, up to 51% of the protein concentration. For *Re*Ohy, the FAD seems to have dissociated completely, indicating particularly low affinity. Indeed, *Re*Ohy required FAD supplementation for product formation. However, for the other Ohys, FAD supplementation did not improve conversions, despite also containing apo‐enzyme. Reductive conditions did clearly improve product yields, for *Re*Ohy at least 12‐fold, emphasizing the important role of the redox state of the cofactor. Although *Sn*Ohy was the most active enzyme at low oleic acid loadings, *Em*Ohy achieved higher product yields at increased oleic acid loading (10 mM). Presumably, this is due to its higher stability, as demonstrated by thermal shift assays: *Em*Ohy showed the highest apparent melting temperature of 54 °C. At higher substrate loading and longer reaction times, either deactivation of the Ohys or limitations in substrate availability occur. In conclusion, in this side‐by‐side biochemical study, *Em*Ohy was identified as a preferred oleate hydratase for further research compared to *Sn*Ohy, *Re*Ohy, and *Rp*Ohy: it shows relatively good expression and FAD affinity, and relatively high stability and activity at increased substrate loading and reaction times. The results provide a good basis for diverse avenues, like protein engineering, studying the phase behavior of the fatty acids, and immobilization, and experiments in these directions are currently underway in our laboratories.

## 4 Experimental Section

### 4.1 Enzyme Expression and Purification

For expression of *Em*Ohy and *Rp*Ohy (pBAD/HisA vector) in *E. coli* TOP10 cells, a preculture of 10 mL LB medium with 100 µg/mL ampicillin was inoculated with a colony from an LB‐agar plate. The preculture was grown overnight at 37 °C and 180 rpm. 5 mL of preculture was added to 500 mL of sterilized TB medium with 100 µg/mL ampicillin and grown again at 37 °C and 180 rpm until an OD_600_ of 0.6–0.8 was reached. Expression was induced by the addition of 0.2% (13.3 mM) L‐arabinose, and growth was resumed at 25 °C and 180 rpm for 15–20 h. To harvest the cells, the culture was centrifuged (7000 × g, 10 min, 4 °C) and the cell pellet was washed with 50 mM Tris‐HCl with 500 mM NaCl, pH 8.0, and centrifuged again (7000 × g, 10 min, 4 °C) before storage at −70 °C. Expression of *Sn*Ohy and *Re*Ohy (on pET28a(+) vector) was performed identically, except that the *E. coli* strain was BL21(DE3) instead of TOP10, the antibiotic was kanamycin (50 µg/mL) instead of ampicillin, and the inducer was IPTG (0.5 mM final concentration) instead of arabinose.

For cell lysis and purification, pellets were resuspended in 50 mM Tris‐HCl, 500 mM NaCl, pH 8.0, containing 1 mM PMSF, 10 mM MgCl_2_, 2 mM CaCl_2_, 0.02 mg/mL DNase, and 0.2 mg/mL lysozyme and shaken on a horizontal shaker for 30 min at room temperature. Cell lysis was done by sonication (Branson Sonifier 450) for 5 min, 40% duty cycle, and output 3.5. Insoluble debris was separated by centrifugation (20,000 rpm, 40 min, 4 °C), and the supernatant was 0.45 µm filtered. For purification by gravity column, the supernatant was loaded onto a Fast Flow nickel Sepharose column pre‐equilibrated with Buffer A (50 mM Tris‐HCl pH 8.0, 500 mM NaCl, 40 mM imidazole). The His‐tagged protein was eluted with elution buffer (50 mM Tris‐HCl pH 8.0, 500 mM NaCl, 400 mM imidazole). Lastly, eluted fractions were desalted using an EconoPac 10‐DG (Bio‐Rad) pre‐equilibrated with 50 mM KP_i_, pH 8.0. The purity of each protein sample was analyzed and verified by SDS‐PAGE and quantified by bichinchonic acid (BCA) assay. Purified protein aliquots were flash‐frozen and stored at −70 °C.

### 4.2 SEC‐MALS Analysis

Purified Ohy samples (concentrations: 1.2 mg/mL (*Em*Ohy), 7.8 mg/mL (*Sn*Ohy), 4.8 mg/mL (*Re*Ohy), and 3.0 mg/mL (*Rp*Ohy)) were centrifuged to remove aggregates (14000xg, 4 °C, 10 min), and 50 µL was transferred to a chromatography vial. The proteins were analyzed by SEC using an HPLC unit (1260 Infinity II, Agilent) with a Superdex200 Increase 10/300 GL column (Cytiva), running in series with an online ultraviolet (UV) detector (1260 Infinity II VWD, Agilent), an eight‐angle static light scattering detector (DAWN HELEOS 8+, Wyatt Technology) and a refractometer (Optilab T‐rEX, Wyatt Technology). The running buffer was 50 mM KP_i_, pH 8.0 with 100 mM NaCl. The molecular weight was calculated based on the measured Rayleigh scattering at different angles and the established differential refractive index increment of value of 0.185 mL/g for proteins in solution with respect to the change in protein concentration (dn/dc) using ASTRA software (Wyatt Technology, version 7.3.1).

### 4.3 UV–Vis Spectroscopy

Purified Ohy samples were centrifuged to remove aggregates (14000 × g, 10 min, 4 °C) and their UV–Vis spectra were recorded on a spectrophotometer (Agilent Cary 60). Denaturation was performed by adding sodium dodecyl sulfate (SDS) to a concentration of 0.2% from a 10% stock and heating at 95 °C for 10 min. Samples were centrifuged again (14000 × g, 10 min, 4 °C), and the spectrum of the supernatant with released FAD was recorded. For determination of the FAD occupancy, the FAD concentration was calculated using its extinction coefficient at 450 nm (ε_450_ = 11,300 M^−1^ cm^−1^) [35].

### 4.4 Thermal Shift Assays

For the ThermoFluor assay, purified Ohy stocks were diluted to 1.1 mg/mL in 50 mM KP_i_, pH 7.6. 2 µL of SYPRO Orange (250x) was added to 18 µL of pure Ohy solution (final Ohy concentration 1 mg/mL, SYPRO Orange 25×) in qPCR tubes with optically clear lids. Samples were transferred to a real‐time PCR machine (Analytik Jena, qTower^3^) with a temperature program 25–85 °C at a heating rate of 1 °C/min and analyzed with the JOE filter (excitation 515 nm, emission 545 nm) at gain 3/10. For the ThermoFAD assay, purified Ohy stocks (20 µL, 3.5 mg/mL) were transferred to qPCR tubes with optically clear lids. Samples were transferred to a real‐time PCR machine (Analytik Jena, qTower^3^) with a temperature program 25–85 °C at a heating rate of 1 °C/min and analyzed with the FAM filter (excitation 470 nm, emission 520 nm) at gain 10/10. For data analysis of both assays, raw data were smoothed using a second‐order polynomial with four neighbors (Savitzky–Golay filter, GraphPad Prism).

### 4.5 Biotransformations

For 1 mM reactions, biotransformations were performed with 100 mM KP_i_, pH 6.0, 7.0, or 8.0 containing 1 µM purified Ohy (concentration with respect to Ohy *monomer*, i.e., number of active sites) and 0 or 4 µM FAD in a total volume of 475 µL, in a 1.5 mL Eppendorf tube. Reactions were initiated by addition of 25 µL of 20 mM OA in DMSO for a total reaction volume of 500 µL with final concentrations of 1 mM OA and 5% v/v DMSO. Reaction samples were incubated at 25 °C with 1000 rpm orbital shaking for 10 min. For reactions under reductive conditions, these conditions were achieved by dissolving 2 mM dithionite (in its sodium salt form) in the buffer and performing the reactions in an anaerobic glove box (MBRAUN). Reactions were terminated by addition of 40 µL 1 M HCl. For 10 mM reactions, biotransformations were performed with 50 mM PIPES, pH 7.0, containing 1 µM purified Ohy (concentration with respect to Ohy *monomer*, i.e., number of active sites) and 4 µM FAD in a total volume of 475 µL, in a 1.5 mL Eppendorf tube. Reactions were initiated by addition of 25 µL of 200 mM OA in DMSO for a total reaction volume of 500 µL with final concentrations of 10 mM OA and 5% v/v DMSO. Reaction samples were incubated at 25 °C with 1000 rpm orbital shaking for 10 min, 2, or 24 h. For reactions under reductive conditions, these conditions were achieved by dissolving 2 mM dithionite (in its sodium salt form) in the buffer and performing the reactions in a glove box. Reactions were terminated by the addition of 40 µL 1 M HCl.

### 4.6 GC‐FID Analysis

Analysis of 1 mM reactions was done as follows: to the acidified reaction samples was added 500 µL of EtOAc containing 0.5 mM *n*‐dodecane as an internal standard, and samples were vortexed for 30 s. Samples were centrifuged briefly, and the extract was dried over MgSO_4_. Subsequently, 25 µL was added to 75 µL of derivatization reagent (BSTFA with 1% TMCS: pyridine, 1: 1) in glass chromatography vials. These were incubated at 60 °C for 1.5 h for silylation before their contents were transferred into plastic 0.3 mL chromatography vials. GC measurements were performed on a Shimadzu type GC‐2014 with a CP‐Sil5 CB column (25 m × 0.25 mm × 1.2 µm) with N_2_ (flow rate 30 mL min^−1^) as carrier gas. Injector and detector (FID) temperatures were 340 and 360 °C, respectively. 1 µL was injected at a split ratio of 100:1. The oven program started at 165 °C (3 min hold) with a 15 °C/min ramp until 345 °C (3 min hold). Analysis of 10 mM reactions was done as follows: to the acidified reaction samples was added 500 µL of EtOAc containing 5 mM decanoic acid methyl ester (DAME) as an internal standard, and samples were vortexed for 2 min. The extract was centrifuged briefly, dried over MgSO_4_, and transferred to a 0.3 mL chromatography vial. GC measurements were performed on a Shimadzu type GC‐2010 Pro with a MET‐Biodiesel capillary GC column (13 m × 0.32 mm, d_f_ 0.10 µm – the 2 m × 0.53 mm I.D. guard was removed) from Merck, and H_2_ as carrier gas (Parker 40H‐MD Carrier generator, flow rate: 70 cm s^−1^). Injector and detector temperatures were 270 and 350 °C, respectively. One microliter was injected at a split ratio of 60:1. The oven program started at 100 °C (1 min hold) with a 30 °C/min ramp until 340 °C (1 min hold).

## Funding

This work was supported by the Dutch Research Council (NWO) domain Applied and Engineering Sciences under the Open Technology Programme grant 20781 (project name EnzIm2Flow).

## Conflicts of Interest

The authors declare no conflicts of interest.

## Supporting information

Supplementary Material

## Data Availability

The data underlying this publication is deposited in the 4TU. ResearchData repository (https://doi.org/10.4121/bf9e1512‐eacd‐4001‐8430‐522f07ee61dd).
