# Supplementary material for: A Comparative Biochemical Study of Oleate Hydratases
Source: Chembiochem. 2026 Jul 30;27(15):e70487. doi: 10.1002/cbic.70487 (PMC13422127; doi:10.1002/cbic.70487)
Supplement: Supplementary file 1 — Supplementary Material [file CBIC-27-e70487-s001.pdf]

ChemBioChem

Supporting Information

A Comparative Biochemical Study of Oleate Hydratases

Maxim van Delft, Alejandro Gran Scheuch, Ulf Hanefeld and Peter-Leon Hagedoorn

Table of Contents

Supplementary Figures 3

Figure S1: SDS-PAGE analysis of the purified oleate hydratases 3

Figure S2: Multiple sequence alignment of the oleate hydratases 4

Figure S3: Effect of FAD supplementation on product yields of the Ohys in biotransformations 4

Figure S4: First-order derivatives of Ohy melting curves as measured by thermal shift assays 5

Figure S5: GC calibration curves of silylated oleic acid and 10-HSA 5

Figure S6: Chromatograms of silylated oleic acid and 10-HSA 5

Figure S7: GC calibration curves of oleic acid and 10-HSA 5

Figure S8: Chromatograms of pure oleic acid and 10-HSA 6

Supplementary Tables 7

Table S1: Amino acid percent identity matrix of the oleate hydratases under study 7

Table S2: Expression yields of the oleate hydratases 7

Supplementary experimental details 8

Supplementary Figures

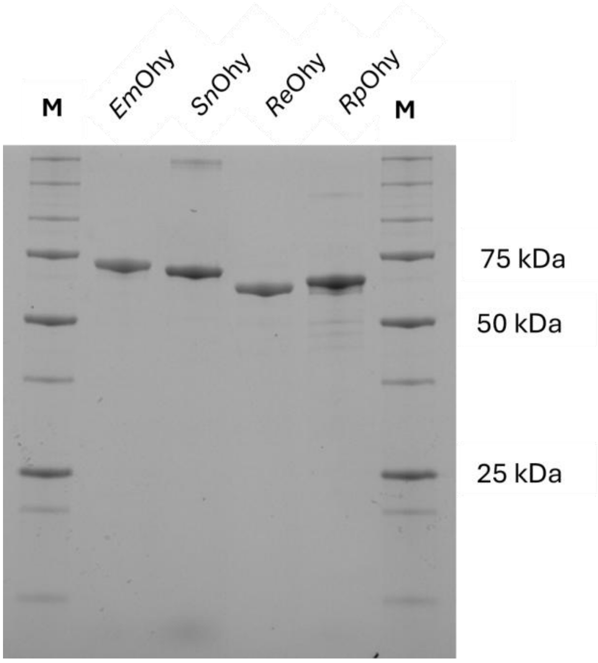

*Figure S1: SDS-PAGE analysis of the purified oleate hydratases. Theoretical MW (including tag): 78 kDa (*EmOhy*), 74 kDa (*SnOhy*), 67 kDa (*ReOhy*), 72 kDa (*RpOhy*). M: protein marker.*

```
ReOhy      --------------------------------------------MLGRYPYRDSSKERNP 16
RpOhy      -----------------------------------------MYYSSGNYEAFA---RPRK 16
EmOhy      MNPITSKFDKVLNASSEYGHVNHEPDSSKEQQRNTPQKSMPFSDQIGNYQ-RN---KGIP 56
SnOhy      -------MEEV---SYPKAGPSIEANVGDGHWRKGPSDTLPPPDTVGPYM-RN---RPLP 46
                                                 * *       .

ReOhy      MSSNLSHKAYMIGAGIGNLSAAVYLIRDGEWNGEDITIMGLD-MHG-ANDGESAATFQHQ 74
RpOhy      PEGVENKTAWFVGAGLTSMASAVFMIRDGQLSGDKITILERLDLPGGALDGI-------- 68
EmOhy      VQSYDNSKIYIIGSGIAGMSAAYYFIRDGHVPAKNITFLEQLHIDGGSLDGA-------- 108
SnOhy      VDQVEGRKAWIIGSGIAGLASAFYLIRDGRMKGQDITILDAVGTPGGSLDGS-------- 98
            .   . . :::*:*: .:::* ::****.  ...**::      * : **

ReOhy      YGHRELGNDAGFINRGGRMLNEETYENLWDILSAVPSLDNPGK-SVTDDILDFDHAHPTH 133
RpOhy      -----KKPDKGFVIRGGREME-DHMECLWDLFRTIPSLEVD--GSVLDEFYWLNKDDPNY 120
EmOhy      -----GNPTDGYIIRGGREMD-MTYENLWDMFQDIPALEMPAPYSVLDEYRLINDNDSNY 162
SnOhy      -----GNAEDGYLIRGGREMN-WNYDHFWDLFQDIPALEYPSPYSVLDEYRAVNDNDPNW 152
                     *:: **** ::    : :**::  :*:*:     ** *:   .:. . .

ReOhy      DVARLIDRDGIRNKGENDYKHMQFDNKDRYLLTKLMTMPESDEAKLDDISIEQWFEDTPH 193
RpOhy      SLNRVTHRQGEEFVTN---NEFGLSEKAQKELVKVF---LASREEMEDKRIDEIFGE--E 172
EmOhy      SKARLINNKGEI-KDF---SKFGLNKMDQLAIIRLL---LKNKEELDDLTIEDYFSE--S 213
SnOhy      SKSRLMHKQGQI-RDF---STLGLSSAHQWELIKLL---LKRKEDLDDITIEQYFSD--S 203
           .  *: ...*         . : :..  :  : :::      . .::*  *:: * :

ReOhy      FFTTNFWYMWETTFAFKRVSSAMELRRYMNRMILEFSRIQTLAGVTRSPYNQYESIILPM 253
RpOhy      FLSSNFWLYWRTMFAFENWHSALELKLYLHRFVHHIGGLPDLSALKFTKYNQYESLVLPM 232
EmOhy      FLKSNFWTFWRTMFAFENWHSLLELKLYMHRFLHAIDGLNDLSSLVFPKYNQYDTFVTPL 273
SnOhy      FLETNFWYLWRSMFAFQNWQSLLEVKLYMHRFLDAIDGLTDMSALVFPKYNQYDSFVVPL 263
           *: :***  *.: ***:.  * :*:: *::*::  :. :  ::.:    ****:::: *:

ReOhy      RTFLEGKGVKFVNELKITEFVFKDTPLRDEIIVTGLDYENVRTGEKGRIDVAEGDFVFDT 313
RpOhy      YRWLLDQGVRFEFSTEVTDIDFVFDGD--RKQATRI--HWTKGGVPGGVDLGPDDLVLAT 288
EmOhy      RKFLQEKGVNIHLNTLVKDLDIHINTE--GKVVEGI--ITEQDGKEVKIPVGKNDYVIVT 329
SnOhy      VNYLKGQGVNVEFGTRVYDLDMTDNNG--ERTVTSI--LAKVDGRDQKIDIGAKDVVFAL 319
             :*  :**..     : :: :          .  :       *    : :.  * *:

ReOhy      NGSITDSSSIGDLDTPIV-----EDMRYAPSALLWKQATEHFYDLGNPDKFFGDRAQSEW 368
RpOhy      IGSLTENSDDGTHHNAAR-----LDEGPAPAWDLWRRIAAKHPSFGRPEVFCGDISKTKW 343
EmOhy      TGSMTEDTFYGNNKTAPIIGIDNSTSGQSAGWKLWKNLAAKSEIFGKPEKFCSNIEKSAW 389
SnOhy      TGSMTEGTAYGDLDTAPDLTRATTPPGDSSDWALWQNLAKKSHVFGKPEKFCGQPSRSMW 379
            **:*:.:  *  ..             :    **:.  : :   :*.*: * .:  :: *

ReOhy      TSFTVTTSSHELINEISRITKQLP------GNALNTFVDSNVLLSIVVHHQPHYHAQKEN 422
RpOhy      ESATVTTIGPEIPRYIKKIAKRDPFSGNIVTGGIVTAKDSSWLLSWTVNRQPHFKAQAPD 403
EmOhy      ESATLTCKPSALIDKLKEYSVNDPYSGKTVTGGIITITDSNWLMSFTCNRQPHFPEQPDD 449
SnOhy      ESATLTCKPSPLTERLKDLSINDPYSGKTVTGGIITFTDSNWVLSFTCNRQPHFPTQPDD 439
            * *:*     :   :.  : . *       ..: *  **. ::* . ::***:  *  :

ReOhy      EGVFWGYCLFPRKDGDYVKKPFIEMTGREMLEETLGHLEALDESGALAARRQEIMDSVVN 482
RpOhy      EIVVWVYGLFVDVPGDFTGKTMQESTGEEITQEWLYHLGVPVEDIPELA------ANGAK 457
EmOhy      VLVLWVYALFMDKEGNYIKKTMLECTGDEILAELCYHLGIED--QLENV------QKNTI 501
SnOhy      VLVLWVYALVMDSKGNHVLKPMPECTGREILAELCYHLGIVD--QVDEV------ARQTK 491
             *.* * *.    *:.  * : * ** *:  *   **          .         .

ReOhy      SIPSHMPYASALFNRRAVGDRPLVVPKHSKNLAFISQFAELPFDMVFTEQYSVRCAQVAV 542
RpOhy      TVPVMMPYVTSFFMPRTAGDRPDVVPEGAVNFAFIGQFAETTRDTIFTTEYSVRTAMEAA 517
EmOhy      VRTAFMPYITSMFMPRAKGDRPRVVPEGCKNLGLVGQFVETNNDVVFTMESSVRTARIAV 561
SnOhy      VRLALMPFITAQFMPRAAGDRPRVVPAGCTNLALLGQFVETSNDIIFTMESSVRTARIGV 551
                **: :: *  *: **** ***  . *:.::.**.*   * :** : *** *  ..

ReOhy      YKFLGIPEDKLTKMHHYEKDPKVLAKAAVTMFR--------------------------- 575
RpOhy      YQLLGIDRGVPE-VFNSTYDLRFLLEATARLRDGEEVELPGPKFVGNRIIKHL-DHTQIG 575
EmOhy      YKLLNLNKQVPD-INPLQYDIRHLLKAAKTLNDDK-------PFVGEGLLRKVLKGTYFE 613
SnOhy      YTLLGLRKQVAD-ISPTQYDVRNLIKGARALNNNE-------PFMGERLLHRLLDNTYFA 603
           * :*.: .     :     * : * :.:  :

ReOhy      -----------------------------------------------------       575
RpOhy      QLLTDFGVIPELDGTT------KRVRADDAGA---------------------       601
EmOhy      HVLPAG---AAEEEEHES-----------FIAEHVNKFREWVKGIRG------       646
SnOhy      HILPPL---PAGDGGSSDQAASSRMKANHTAAAALGAVSDWIHHVRDKLKPGA       653
```

Figure S2: Multiple sequence alignment of the oleate hydratases. Made with Clustal 0(1.2.4).

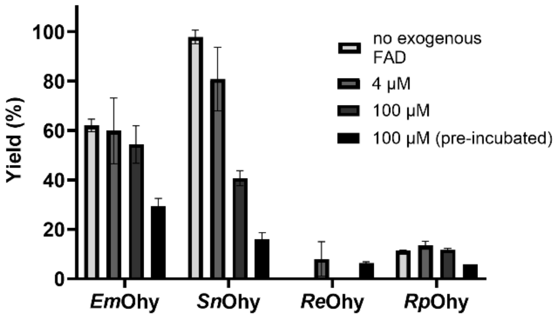

Figure S3: Effect of FAD supplementation on product yields of the Ohys in biotransformations. Reaction conditions: 1.0 mM OA, 1.0 µM Ohy, 0, 4.0 or 100 µM (pre-incubated) FAD, 5.0% v/v DMSO, in 100 mM KP_i, pH 7.0, total volume 500 µL; 25 °C, 1000 rpm orbital shaking, 10 min. For pre-incubation conditions, 10 µM of Ohy was incubated with 1.0 mM FAD at room temperature for 2 h. Each bar represents the mean of triplicates and error bars are their standard deviation.

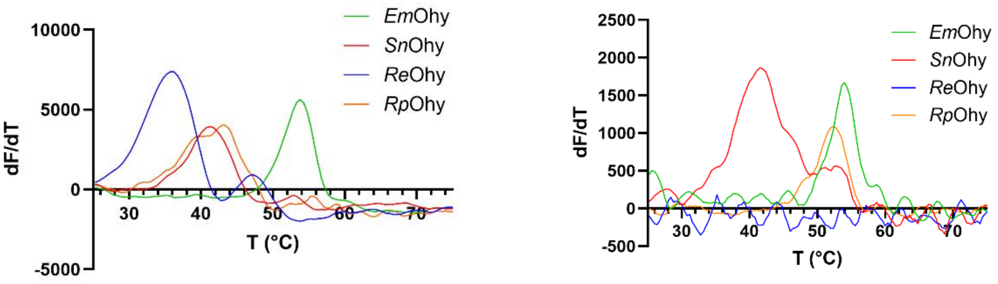

Figure S4: First-order derivatives of Ohy melting curves as measured by thermal shift assays. The left graph corresponds to ThermoFluor assay, the right graph to ThermoFAD assay.

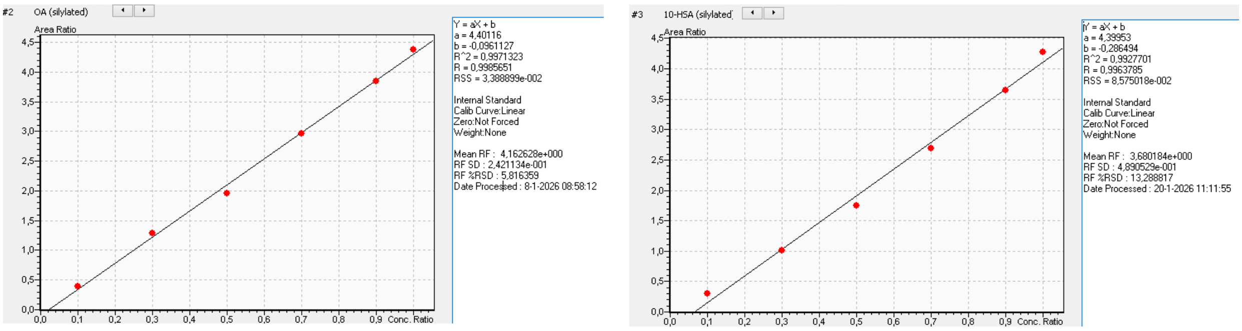

Figure S5: GC calibration curves of silylated oleic acid and 10-HSA. The left curve represents silylated oleic acid, the right curve silylated 10-HSA. n-dodecane was used as internal standard, N_2 as carrier gas.

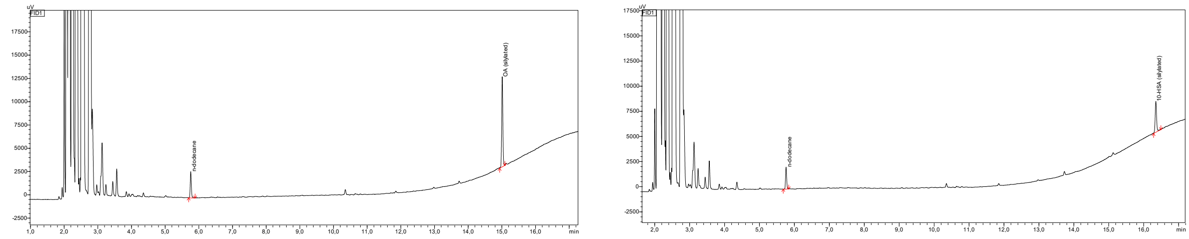

Figure S6: Chromatograms of silylated oleic acid and 10-HSA. The left chromatogram represents silylated oleic acid (r_t = 15.0 min), the right chromatogram silylated 10-HSA (r_t = 16.4 min). n-dodecane (r_t = 5.8 min) was used as internal standard, N_2 as carrier gas.

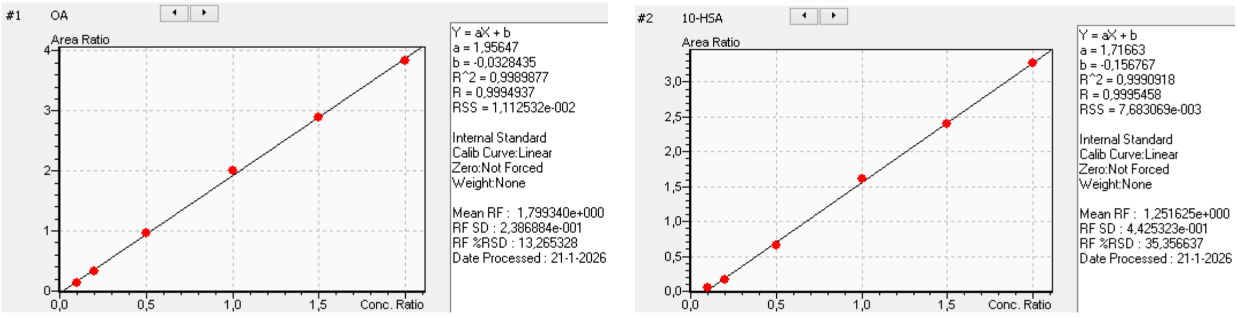

Figure S7: GC calibration curves of oleic acid and 10-HSA. The left curve represents oleic acid, the right curve 10-HSA. Decanoic acid methyl ester (DAME) was used as internal standard, H_2 as carrier gas.

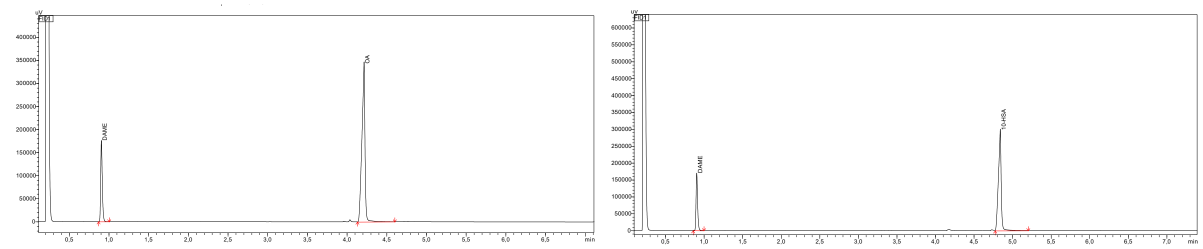

Figure S8: Chromatograms of pure oleic acid and 10-HSA. The left chromatogram represents oleic acid (r_t = 4.2 min), the right chromatogram 10-HSA (r_t = 4.8 min). DAME (r_t = 0.9 min) was used as internal standard, H_2 as carrier gas.

Supplementary Tables

Table S1: Amino acid percent identity matrix of the oleate hydratases under study. Data obtained by multiple sequence alignment with Clustal 0(1.2.4).

	***Em*Ohy**	***Sn*Ohy**	***Re*Ohy**	***Rp*Ohy**
***Em*Ohy**	100	57.7	33.5	42.8
***Sn*Ohy**	57.7	100	33.1	43.4
***Re*Ohy**	33.5	33.1	100	33.3
***Rp*Ohy**	42.8	43.4	33.3	100

Table S2: Expression yields of the oleate hydratases.

**Ohy**	**Codon optimized?**	**Vector/strain**	**No. of purifications**	**Expression yield ($mg/L_{culture}$)**
***Em*Ohy**	no	pBAD/TOP10	5	28 ± 11
	yes	pET/BL21(DE3)	2	9 ± 2
***Sn*Ohy**	yes	pET/BL21(DE3)	2	99 ± 14
***Re*Ohy**	yes	pBAD/TOP10	1	35
	yes	pET/BL21(DE3)	2	63 ± 6
***Rp*Ohy**	yes	pBAD/TOP10	2	54 ± 16
	yes	pET/BL21(DE3)	1	39

Supplementary experimental details

Chemicals

Chemicals were obtained from commercial suppliers (Sigma-Aldrich/Merck, ThermoFisher Scientific) in the highest available purity. Pure *(R)*-10-hydroxystearic acid was obtained from dr. Martin Schürmann (InnoSyn).

Sequences

His-tags are underlined in both DNA and protein sequences; tag sequences are separated from gene/protein sequences by a vertical line. Gene/protein accession numbers are given in brackets.

*Em*Ohy

DNA sequence (GQ144652)

ATGGGGGGTTCT<u>CATCATCATCATCATCAT</u>GGTATGCTAGCATGACTGGTGGACAGCAAATGGGTCGGGATCTGTACGACGAT
GACGATAAGGACGATGGGGATCCGAGCTCGA|ATGAACCCAATAACTTCAAAATTTGACAAAGTACTTAATGCTTCTTCCGAATA
CGGACATGTAAACCATGAACCGGATTCCAGTAAAGAACAGCAACGAAACACCCCGCAAAAATCAATGCCCTTTTCTGATCAG
ATTGGAAATTATCAGAGAAACAAAGGGATTCCTGTACAATCATATGACAATAGTAAGATTTACATTATAGGCAGTGGAATCGCAGG
TATGTCGGCAGCTTATTATTTTATACGCGATGGGCATGTTCCTGCAAAAAACATCACCTTCTTGGAACAATTGCATATCGATGGC
GGTTCATTAGATGGTGCCGGAAATCCGACAGACGGCTATATTATCCGTGGCGGTCGTGAAATGGACATGACGTACGAAAATCT
TTGGGATATGTTTCAGGATATACCTGCCTTAGAAATGCCTGCTCCTTACAGTGTACTGGACGAATACAGATTAATTAATGATAACG
ACTCCAATTATTCTAAAGCCCGGTTAATCAACAATAAAGGTGAGATAAAAGACTTTAGCAAGTTCGGCCTAAATAAAATGGACCA
GTTAGCTATTATCAGATTACTTCTGAAAAATAAAGAAGAACTGGACGATTTAACCATTGAGGATTACTTCAGCGAATCCTTCCTGA
AAAGTAATTTCTGGACTTTTTGGAGAACGATGTTTGCCTTTGAAAACTGGCATAGCTTATTGGAACTGAAACTTTACATGCACCGT
TTCCTTCACGCCATAGACGGACTGAACGATCTGTCTTCACTGGTATTCCCTAAATACAACCAATACGACACCTTCGTAACTCCT
CTGCGCAAATTCCTTCAGGAAAAAGGTGTTAATATCCACCTGAACACTCTGGTAAAAGATCTGGATATCCACATCAATACCGAA
GGAAAAGTTGTAGAAGGAATTATCACCGAACAGGATGGTAAGGAAGTAAAAATCCCTGTTGGTAAAAATGACTATGTCATTGTAA
CTACAGGTTCCATGACGGAAGATACCTTCTACGGAAATAATAAAACTGCTCCTATTATTGGCATAGACAACAGCACAAGCGGAC
AAAGTGCCGGATGGAAGTTGTGGAAAAATCTGGCTGCAAAATCAGAAATTTTTGGGAAACCAGAGAAATTCTGCAGCAATATCG
AGAAATCTGCATGGGAATCTGCAACGCTAACCTGTAAACCTTCAGCCCTTATCGACAAGCTGAAAGAATACTCTGTTAACGATC
CATATTCCGGAAAAACTGTTACCGGCGGTATTATTACCATTACAGATTCCAACTGGCTGATGAGTTTCACCTGCAACAGACAGC
CACACTTCCCGGAACAGCCGGATGATGTACTGGTACTTTGGGTATATGCCTTATTCATGGACAAAGAGGGAAACTATATCAAAA
AAACAATGCTGGAATGTACAGGAGATGAAATTCTTGCAGAATTATGCTACCATTTAGGTATTGAAGATCAGCTGGAAAATGTACAG
AAAAATACAATTGTAAGAACTGCATTCATGCCCTATATAACTTCTATGTTTATGCCAAGAGCTAAAGGCGATCGCCCTAGAGTAGT
GCCTGAAGGCTGTAAAAATCTGGGACTGGTAGGTCAGTTTGTAGAAACCAATAATGATGTGGTATTTACAATGGAAAGCTCTGTA
AGAACAGCGAGAATTGCTGTCTACAAATTACTAAACCTCAACAAACAGGTTCCTGATATCAATCCTTTACAGTATGATATCCGAC
ATCTGCTAAAAGCAGCAAAAACACTGAATGATGACAAACCATTTGTAGGTGAAGGCTTGTTGAGAAAAGTCCTTAAAGGAACTTA
CTTTGAACATGTGTTACCTGCCGGTGCAGCAGAGGAAGAAGAACATGAATCCTTTATCGCTGAACATGTAAATAAGTTCAGAGA
ATGGGTAAAAGGAATAAGAGGATAA

Protein sequence (ACT54545.1)

MGGS<u>HHHHHH</u>GMASMTGGQQMGRDLYDDDDKDRWGSELE|MNPITSKFDKVLNASSEYGHVNHEPDSSKEQQRNTPQKS
MPFSDQIGNYQRNKGIPVQSYDNSKIYIIGSGIAGMSAAYYFIRDGHVPAKNITFLEQLHIDGGSLDGAGNPTDGYIIRGGREMDM
TYENLWDMFQDIPALEMPAPYSVLDEYRLINDNDSNYSKARLINNKGEIKDFSKFGLNKMDQLAIIRLLLKNKEELDDLTIEDYFSE
SFLKSNFWTFWRTMFAFENWHSLLELKLYMHRFLHAIDGLNDLSSLVFPKYNQYDTFVTPLRKFLQEKGVNIHLNTLVKDLDIHIN
TEGKVVEGIITEQDGKEVKIPVGKNDYVIVTTGSMTEDTFYGNNKTAPIIGIDNSTSGQSAGWKLWKNLAAKSEIFGKPEKFCSNIEK
SAWESATLTCKPSALIDKLKEYSVNDPYSGKTVTGGIITITDSNWLMSFTCNRQPHFPEQPDDVLVLWVYALFMDKEGNYIKKTML
ECTGDEILAELCYHLGIEDQLENVQKNTIVRTAFMPYITSMFMPRAKGDRPRVVPEGCKNLGLVGQFVETNNDVVFTMESSVRTA
RIAVYKLLNLNKQVPDINPLQYDIRHLLKAAKTLNDDKPFVGEGLLRKVLKGTYFEHVLPAGAAEEEEHESFIAEHVNKFREWVKGI
RG

*Sn*Ohy

DNA sequence (KX162589; codon optimized)

ATGCATCATCATCATCATCATCTCGAC | ATGGAAGAGGTTTCTTACCCTAAGGCCGGTCCTTCTATAGAGGCTAATGTTGGTGACGGTCA
CTGGCGTAAGGGTCCATCTGACACATTACCACCACCAGACACAGTTGGACCCTACATGCGGAATCGACCATTACCTGTAGACCAAGTT
GAGGGACGTAAGGCCTGGATCATCGGTTCGGGTATAGCAGGTCTTGCAAGTGCTTTCTACCTTATCCGTGACGGGAGAATGAAGGGTC
AAGACATCACGATACTCGACGCTGTTGGGACACCCGGCGGGTCACTTGACGGTTCGGGTAATGCAGAGGACGGTTACTTAATCCGAG
GCGGTCGTGAAATGAATTGGAATTACGACCACTTCTGGGACCTTTTCCAAGACATCCCAGCTTTAGAGTACCCTTCACCCTACAGTGTCT
TAGACGAGTACCGAGCAGTTAATGACAATGACCCCAATTGGTCTAAGTCTCGTTTGATGCACAAGCAAGGACAAATCCGTGACTTCAGT
ACATTGGGTCTTTCCTCTGCCCACCAATGGGAGCTCATAAAGCTTTTACTGAAGCGAAAGGAAGACTTAGACGACATAACGATCGAGCA
ATACTTCTCTGACTCATTCCTTGAGACAAATTTCTGGTACCTTTGGCGTTCAATGTTCGCCTTCCAAAATTGGCAAAGTCTTTTAGAGGTCA
AGTTATATATGCACAGATTCTTGGACGCAATCGACGGACTTACTGACATGAGTGCTCTTGTTTTCCCAAAATACAATCAATACGACAGTTT
CGTTGTTCCTTTGGTTAATTACCTCAAGGGTCAAGGTGTTAATGTCGAGTTCGGAACAAGAGTCTACGACTTAGACATGACGGACAATAAT
GGTGAGAGAACGGTCACAAGTATCCTCGCCAAGGTTGACGGTCGTGACCAAAAGATCGACATAGGTGCAAAGGACGTTGTCTTCGCC
CTTACAGGGTCTATGACAGAGGGGACGGCCTACGGAGACTTGGACACAGCCCCTGACTTGACACGTGCAACAACACCACCAGGAGA
CTCCTCAGACTGGGCTCTCTGGCAAAATTTGGCTAAGAAGTCGCACGTATTCGGAAAGCCTGAGAAGTTCTGTGGTCAACCCTCTAGAT
CCATGTGGGAGTCGGCTACTTTAACATGTAAGCCATCGCCCCTCACGGAGCGATTAAAGGACCTCTCCATCAATGACCCTTACTCAGG
AAAGACAGTCACAGGCGGTATCATAACATTCACTGACTCGAATTGGGTTCTTAGTTTCACTTGTAATCGTCAACCACACTTCCCTACACAA
CCAGACGATGTTCTTGTCCTCTGGGTATACGCCTTAGTTATGGACAGTAAGGGAAATCACGTCCTCAAGCCCATGCCCGAGTGTACTGG
TCGTGAGATCTTAGCAGAGTTATGTTACCACTTAGGGATAGTCGACCAGGTCGACGAGGTTGCACGTCAAACGAAGGTTCGTCTCGCCT
TGATGCCTTTCATAACAGCCCAATTTATGCCCAGAGCAGCCGGCGACCGTCCACGGGTAGTTCCAGCTGGTTGCACGAATTTGGCATT
ACTTGGACAATTCGTTGAGACGTCAAATGACATCATATTCACGATGGAGTCATCCGTCCGTACTGCACGAATAGGTGTATACACGCTCCT
CGGATTACGGAAGCAAGTAGCCGACATAAGTCCTACTCAATACGACGTCCGTAATCTCATAAAGGGCGCACGTGCACTCAATAATAATG
AGCCTTTCATGGGAGAGCGGCTTCTTCACCGGTTACTCGACAATACGTACTTCGCCCACATATTACCGCCATTACCAGCTGGTGACGG
TGGTTCGTCGGACCAAGCTGCCAGTAGCAGAATGAAGGCCAATCACACTGCAGCCGCTGCTCTCGGTGCAGTTTCAGACTGGATCCA
CCACGTCCGTGACAAGCTTAAGCCCGGGGCATAA

Protein sequence (AND01240.1)

MHHHHHHLD | MEEVSYPKAGPSIEANVGDGHWRKGPSDTLPPPDTVGPYMRNRPLPVDQVEGRKAWIIGSGIAGLASAFYLIR
DGRMKGQDITILDAVGTPGGSLDGSGNAEDGYLIRGGREMNWNYDHFWDLFQDIPALEYPSPYSVLDEYRAVNDNDPNWSKS
RLMHKQGQIRDFSTLGLSSAHQWELIKLLLKRKEDLDDITIEQYFSDSFLETNFWYLWRSMFAFQNWQSLLEVKLYMHRFLDAID
GLTDMSALVFPKYNQYDSFVVPLVNYLKGQGVNVEFGTRVYDLDMTDNNGERTVTSILAKVDGRDQKIDIGAKDVVFALTGSMTE
GTAYGDLDTAPDLTRATTPPGDSSDWALWQNLAKKSHVFGKPEKFCGQPSRSMWESATLTCKPSPLTERLKDLSINDPYSGKTVT
GGIITFTDSNWVLSFTCNRQPHFPTQPDDVLVLWVYALVMDSKGNHVLKPMPECTGREILAELCYHLGIVDQVDEVARQTKVRLA
LMPFITAQFMPRAAGDRPRVVPAGCTNLALLGQFVETSNDIIFTMESSVRTARIGVYTLLGLRKQVADISPTQYDVRNLIKGARALN
NNEPFMGERLLHRLLDNTYFAHILPPLPAGDGGSSDQAASSRMKANHTAAAALGAVSDWIHHVRDKLKPG

*Re*Ohy

DNA sequence (NC_012490.1; codon optimized)

ATGCATCATCATCATCATCATCTCGAC | ATGTTGGGTCGTTACCCGTACCGTGACTCTTCTAAAGAACGTAACCCGATGTCTTCTAA
CCTGTCTCACAAAGCGTACATGATCGGCGCGGGCATCGGTAACCTGAGCGCGGCTGTTTATCTGATCCGTGATGGTGAATGG
AACGGTGAAGACATCACCATCATGGGTCTGGACATGCACGGCGCAAACGATGGTGAATCTGCTGCGACTTTCCAGCACCAG
TATGGTCACCGCGAACTGGGTAACGACGCAGGCTTCATTAACCGTGGTGGTCGTATGCTGAACGAAGAAACCTACGAAAACC
TGTGGGACATCCTGTCTGCTGTCCCGTCTCTGGATAATCCGGGCAAATCCGTTACTGATGACATCCTGGATTTCGATCATGCAC
ACCCGACCCACGACGTAGCGCGTCTGATCGATCGTGATGGTATTCGTAACAAAGGTGAAAACGATTACAAACACATGCAGTTC
GACAACAAAGACCGTTATCTGCTGACTAAACTGATGACTATGCCGGAATCTGATGAAGCGAAACTGGATGACATCTCTATCGAA
CAGTGGTTCGAAGATACCCCGCACTTCTTCACCACTAACTTCTGGTACATGTGGGAAACTACCTTTGCTTTCAAACGCGTTTCT
AGCGCGATGGAACTGCGTCGCTATATGAACCGTATGATCCTGGAATTCTCTCGTATCCAGACCCTGGCTGGTGTTACTCGCTC
CCCATACAACCAGTACGAATCTATCATCCTGCCGATGCGCACCTTCCTGGAAGGTAAAGGCGTTAAATTCGTGAACGAACTGA
AAATCACCGAATTCGTGTTCAAAGACACCCCGCTGCGTGATGAAATCATCGTTACCGGTCTGGACTACGAAAACGTTCGTACC
GGTGAAAAAGGCCGCATTGATGTTGCTGAAGGTGACTTCGTTTTCGACACCAACGGTTCCATTACCGACTCTTCCAGCATCGG
TGACCTGGATACCCCGATCGTTGAAGACATGCGCTACGCTCCGTCCGCGCTGCTGTGGAAACAGGCAACCGAACACTTCTA
CGACCTGGGTAACCCGGATAAATTCTTCGGTGACCGCGCACAGTCTGAATGGACCTCTTTCACCGTTACCACTTCTAGCCAC
GAACTGATTAACGAAATCTCCCGTATTACCAAACAGCTGCCGGGTAACGCACTGAACACCTTCGTTGATTCTAACGTTCTGCTG

TCTATCGTAGTTCACCATCAGCCGCATTACCACGCTCAGAAAGAAAACGAAGGTGTGTTCTGGGGTTACTGCCTGTTCCCGCG
TAAAGACGGCGACTACGTTAAAAAACCGTTCATCGAAATGACCGGTCGTGAAATGCTGGAAGAAACCCTGGGTCACCTGGAA
GCTCTGGATGAAAGCGGCGCGCTGGCGGCTCGTCGTCAGGAAATCATGGACTCTGTAGTTAACTCTATCCCGAGCCACATG
CCGTACGCTTCTGCTCTGTTCAACCGTCGTGCGGTTGGCGATCGTCCGCTGGTTGTTCCGAAACACTCCAAAAACCTGGCTT
TCATCAGCCAGTTCGCTGAACTGCCGTTCGACATGGTTTTCACTGAACAGTACTCTGTTCGTTGTGCGCAGGTTGCTGTTTACA
AATTCCTGGGCATCCCGGAAGACAAACTGACCAAAATGCACCACTACGAAAAAGACCCGAAAGTGCTGGCTAAAGCGGCTG
TTACCATGTTCCGTTAA

Protein sequence (WP_020905674.1)

MHHHHHHLD|MLGRYPYRDSSKERNPMSSNLSHKAYMIGAGIGNLSAAVYLIRDGEWNGEDITIMGLDMHGANDGESAATFQ
HQYGHRELGNDAGFINRGGRMLNEETYENLWDILSAVPSLDNPGKSVTDDILDFDHAHPTHDVARLIDRDGIRNKGENDYKHM
QFDNKDRYLLTKLMTMPESDEAKLDDISIEQWFEDTPHFFTTNFWYMWETTFAFKRVSSAMELRRYMNRMILEFSRIQTLAGVTR
SPYNQYESIILPMRTFLEGKGVKFVNELKITEFVFKDTPLRDEIIVTGLDYENVRTGEKGRIDVAEGDFVFDTNGSITDSSSIGDLDTPI
VEDMRYAPSALLWKQATEHFYDLGNPDKFFGDRAQSEWTSFTVTTSSHELINEISRITKQLPGNALNTFVDSNVLLSIVVHHQPHY
HAQKENEGVFWGYCLFPRKDGDYVKKPFIEMTGREMLEETLGHLEALDESGALAARRQEIMDSVVNSIPSHMPYASALFNRRAV
GDRPLVVPKHSKNLAFISQFAELPFDMVFTEQYSVRCAQVAVYKFLGIPEDKLTKMHHYEKDPKVLAKAAVTMFR

*Rp*Ohy

DNA sequence (MN563121; codon optimized)

ATGGGGGGTTCTCATCATCATCATCATCATGGTATGGCTAGCATGACTGGTGGACAGCAAATGGGTCGGGATCTGTACGACGA
TGACGATAAGGATCGATGGGGATCCGAGCTCGAGATCTGCAGC|TACTATTCTTCGGGCAATTATGAGGCGTTCGCGCGTCCT
CGCAAACCCGAGGGCGTAGAGAACAAGACGGCCTGGTTTGTGGGTGCAGGCTTGACGTCGATGGCCAGTGCGGTGTTCAT
GATCCGCGATGGACAACTGAGTGGCGACAAAATTACTATTTTGGAACGTCTGGATCTTCCCGGAGGGGCCCTGGATGGAATT
AAAAAGCCTGACAAGGGATTTGTAATCCGTGGAGGTCGCGAGATGGAAGATCACATGGAATGCCTTTGGGACTTGTTTCGTAC
CATCCCGTCCTTAGAAGTCGATGGTTCAGTCTTGGATGAATTCTATTGGTTAAACAAGGACGACCCTAACTATTCGTTAAATCGC
GTGACACACCGCCAAGGCGAAGAGTTCGTTACCAATAATGAGTTTGGACTTTCCGAAAAGGCACAAAAAGAGTTGGTCAAAGT
TTTCTTGGCGTCTCGTGAAGAAATGGAGGATAAACGTATCGACGAGATTTTCGGGGAAGAATTTTTATCATCAAACTTTTGGCTGT
ACTGGCGTACCATGTTCGCTTTTGAAAACTGGCATAGTGCACTGGAGTTGAAACTGTACCTGCATCGTTTCGTGCATCACATCG
GCGGATTGCCTGATCTTTCCGCCTTGAAATTTACAAAGTACAATCAGTACGAAAGTCTTGTGCTTCCAATGTATCGTTGGTTGTTG
GATCAAGGAGTACGCTTTGAGTTCTCTACCGAGGTCACAGATATCGATTTTGTGTTCGATGGTGACCGCAAGCAGGCCACGCG
TATCCATTGGACAAAAGGAGGTGTGCCAGGCGGAGTTGATTTAGGCCCCGATGATTTAGTCCTGGCAACAATCGGTAGCCTG
ACCGAGAATTCTGACGACGGAACACACCATAATGCCGCGCGTTTAGACGAAGGACCGGCGCCCGCTTGGGACCTGTGGC
GTCGTATTGCCGCGAAACATCCGAGCTTCGGACGCCCCGAGGTTTTCTGTGGAGATATCTCAAAAACAAAGTGGGAATCGGC
TACCGTTACGACGATCGGACCCGAAATCCCGCGCTATATTAAAAAGATCGCGAAGCGTGATCCCTTTTCGGGAAACATTGTCA
CTGGGGGTATCGTGACGGCGAAAGACAGCAGCTGGTTACTTTCATGGACGGTCAACCGTCAACCACATTTCAAGGCACAAG
CGCCAGATGAGATTGTTGTGTGGGTGTATGGTCTGTTCGTAGATGTACCAGGGGATTTTACTGGTAAGACAATGCAGGAGAGTA
CCGGGGAAGAGATCACTCAGGAGTGGTTATACCATCTGGGAGTGCCTGTAGAAGATATCCCCGAGTTAGCGGCAAACGGCG
CAAAAACGGTACCCGTCATGATGCCTTACGTCACGTCATTCTTTATGCCTCGTACAGCTGGGGATCGCCCCGACGTGGTGCC
AGAAGGTGCCGTTAACTTTGCCTTCATTGGTCAGTTCGCTGAGACAACGCGCGATACTATCTTCACGACAGAATACTCAGTGC
GTACAGCCATGGAGGCAGCTTACCAATTGCTGGGCATCGATCGTGGCGTGCCCGAAGTGTTTAACTCTACTTATGATTTACGCT
TCTTGCTGGAGGCTACCGCTCGTTTGCGCGATGGCGAAGAGGTGGAGCTGCCCGGGCCTAAATTTGTAGGCAACCGCATCA
TTAAACACCTTGATCATACACAAATTGGTCAATTATTGACTGACTTTGGCGTCATTCCGGAACTGGACGGTACTACTAAACGTGT
CCGTGCTGATGACGCCGGAGCATGA

Protein sequence (QOV97383.1)

MGGSHHHHHHGMASMTGGQQMGRDLYDDDDKDRWGSELEICS|YYSSGNYEAFARPRKPEGVENKTAWFVGAGLTSMASA
VFMIRDGQLSGDKITILERLDLPGGALDGIKKPDKGFVIRGGREMEDHMECLWDLFRTIPSLEVDGSVLDEFYWLNKDDPNYSLN
RVTHRQGEEFVTNNEFGLSEKAQKELVKVFLASREEMEDKRIDEIFGEEFLSSNFWLYWRTMFAFENWHSALELKLYLHRFVHHI
GGLPDLSALKFTKYNQYESLVLPMYRWLLDQGVRFEFSTEVTDIDFVFDGDRKQATRIHWTKGGVPGGVDLGPDDLVLATIGSLT
ENSDDGTHHNAARLDEGPAPAWDLWRRIAAKHPSFGRPEVFCGDISKTKWESATVTTIGPEIPRYIKKIAKRDPFSGNIVTGGIVTA
KDSSWLLSWTVNRQPHFKAQAPDEIVVWVYGLFVDVPGDFTGKTMQESTGEEITQEWLYHLGVPVEDIPELAANGAKTVPVMM

PYVTSFFMPRTAGDRPDVVPEGAVNFAFIGQFAETTRDTIFTTEYSVRTAMEAAYQLLGIDRGVPEVFNSTYDLRFLLEATARLRDGE
EVELPGPKFVGNRIIKHLDHTQIGQLLTDFGVIPELDGTTKRVRADDAGA
